# Correction: How convincing is a matching Y-chromosome profile?

**DOI:** 10.1371/journal.pgen.1012099

**Published:** 2026-03-30

**Authors:** Mikkel M. Andersen, David J. Balding

Fig 5 is incorrect. The authors have provided a corrected version here.

S2 Table is incorrect. It can be viewed below.

**Fig 5:**
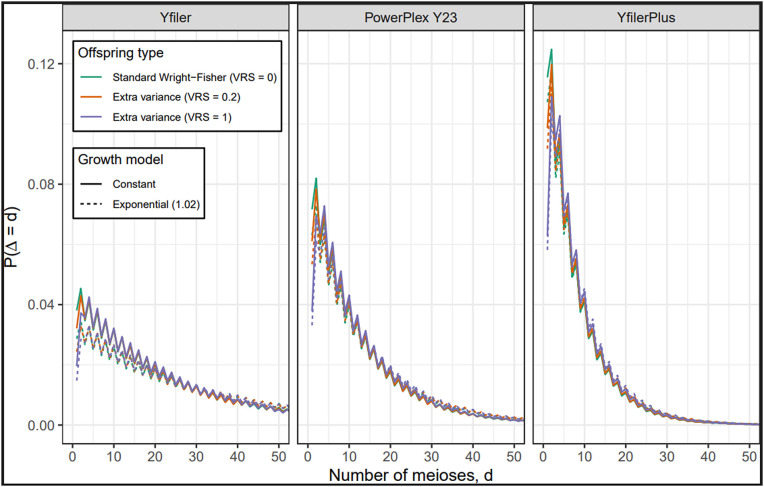
The distribution of Δ, the number of father-son steps between Q and another male in Ω. The distribution is shown for each of three profiling kits, three values of VRS and with and without population growth. See S2 Table for numerical properties of these distributions.

## Supporting information

S2 Table Properties of the distribution of Δ, the number of father-son steps (or meioses) between Q and another male in Ω. These distributions are shown in Fig 5.
(TEX)
