# Supplementary figures and images for: How convincing is a matching Y-chromosome profile?
Source: PLoS Genet. 2017 Nov 3;13(11):e1007028. doi: 10.1371/journal.pgen.1007028 (PMC5669422; doi:10.1371/journal.pgen.1007028)

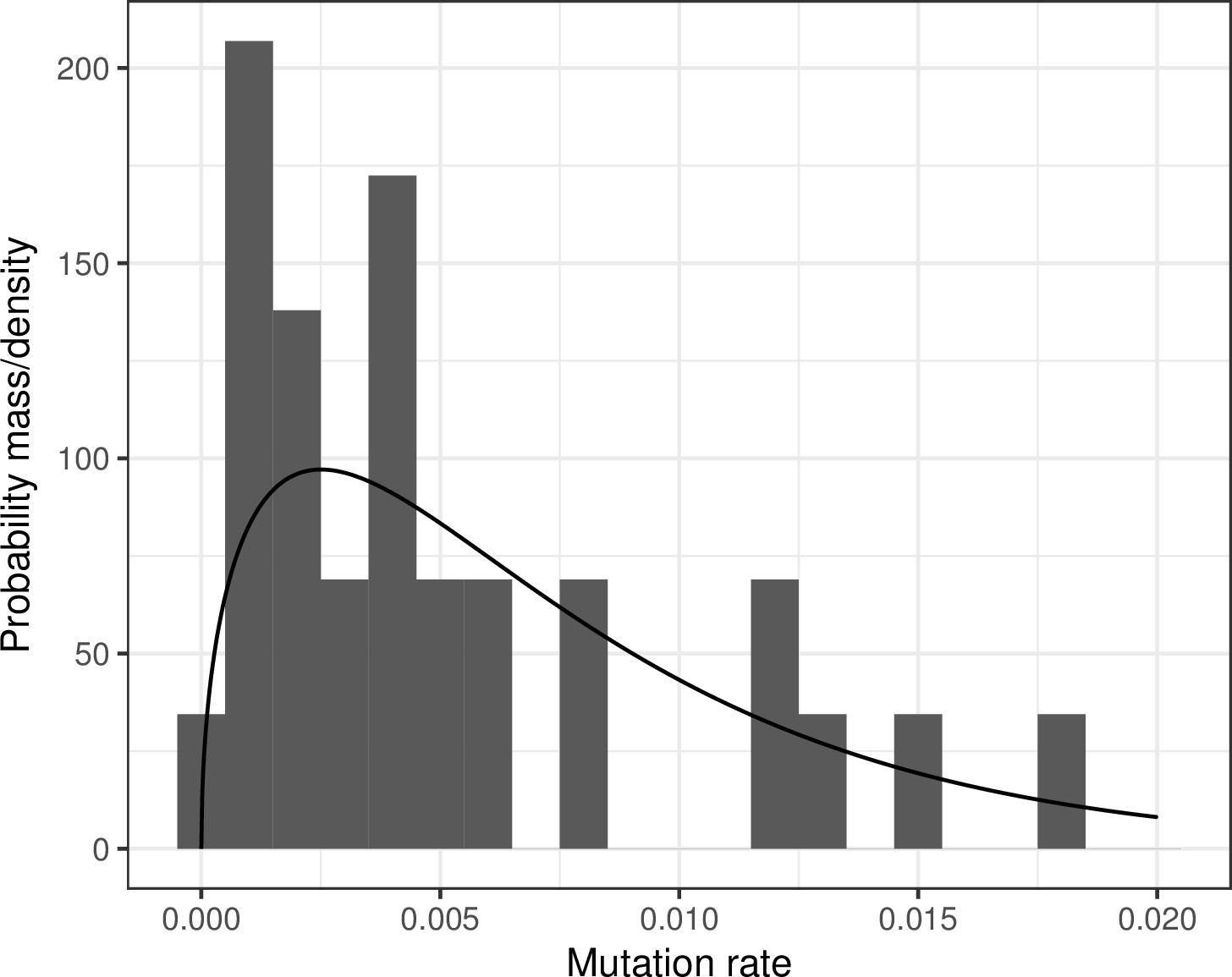

Supplement: S1 Fig — Histogram bars show empirical mutation rates for the 29 loci included in the three Y-STR profiling kits (rates obtained as a ratio of the counts in S1 Table). The two duplicated loci (DYS385 and DYF387S1) are each represented as two loci with the same mutation rate. The curve shows the probability density for the Beta(1.5, 200) prior distribution assumed for each mutation rate. (TIF) [file pgen.1007028.s001.tif]

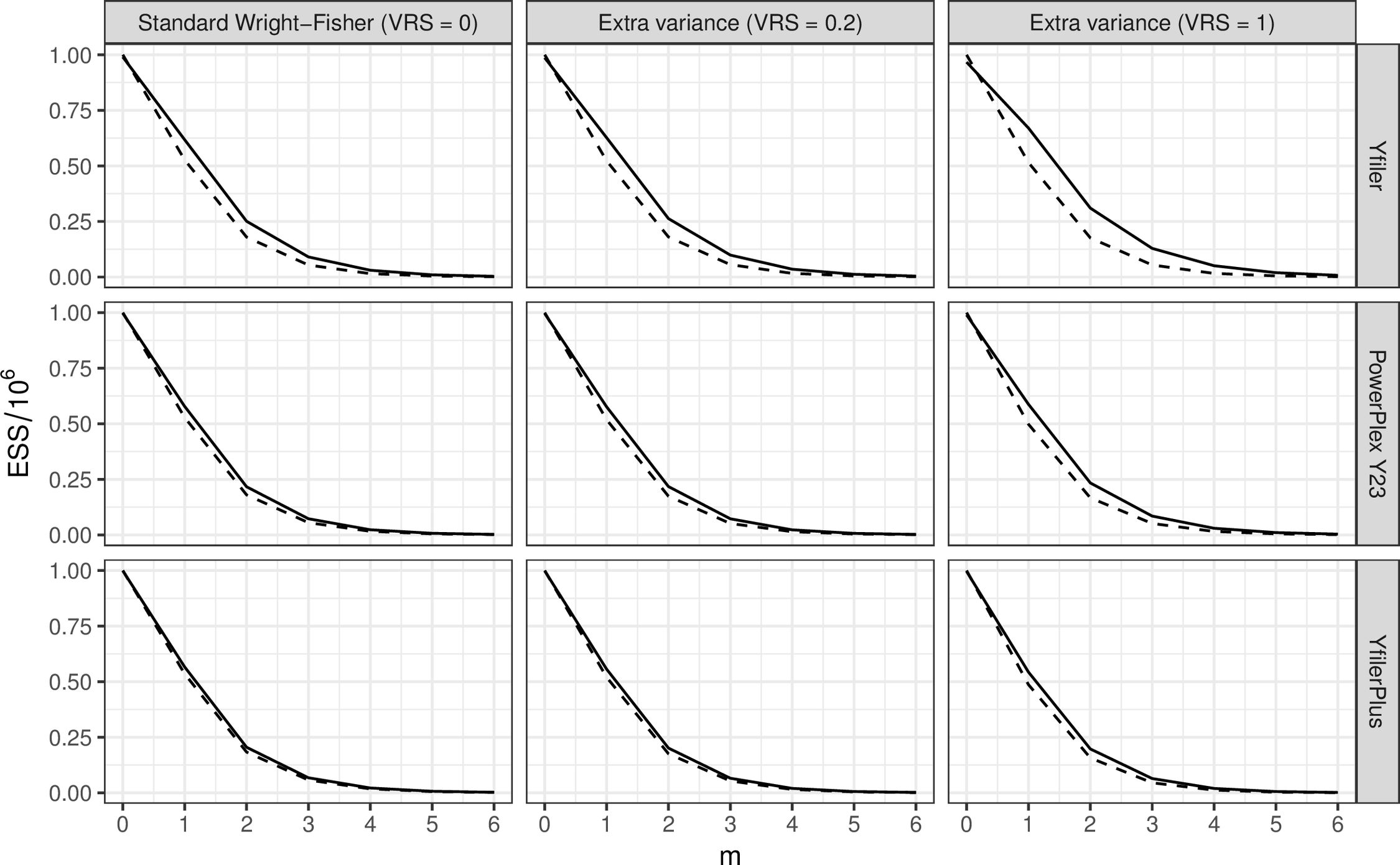

Supplement: S2 Fig — The effective sample size for simulations with constant population size, as a fraction of the 106 simulated |Ω| values, for the importance sampling to approximate distributions for |Ω| conditional on database profile count m, for m from 0 to 6. The database sizes are n = 100 (dashed line) and n = 1,000 (solid line). (TIF) [file pgen.1007028.s002.tif]

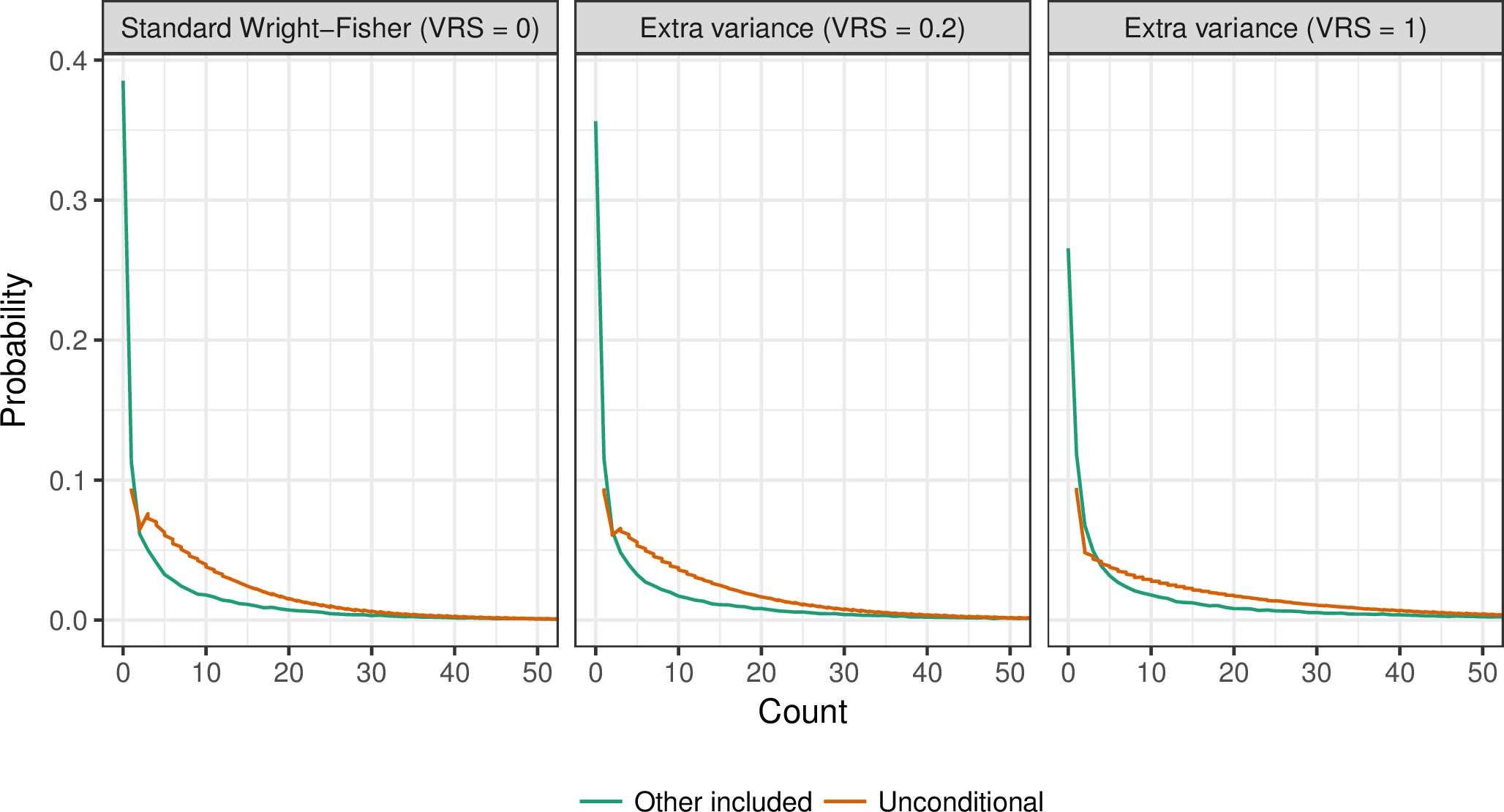

Supplement: S3 Fig — Distribution of the number of Y profiles included in a mixed Yfiler Plus profile arising from two male contributors. The red curve corresponds to profiles that exactly match the profile of one of the contributors. It is the same as the red (“unconditional”) curve in Fig 4 and is included again here for comparison. The green curve corresponds to “other included”: males with a Y profile that consist entirely of alleles within the profiles of the two contributors, and which therefore cannot be excluded from being one of the contributing profiles, but they do not fully match either of the two contributing profiles. (TIF) [file pgen.1007028.s003.tif]
