# Supplementary material for: How convincing is a matching Y-chromosome profile?
Source: PLoS Genet. 2017 Nov 3;13(11):e1007028. doi: 10.1371/journal.pgen.1007028 (PMC5669422; doi:10.1371/journal.pgen.1007028)
Supplement: S1 Text — An approach similar to the one presented for DNA samples with a single male contributor can be applied for DNA samples with multiple male contributors. (PDF) [file pgen.1007028.s013.pdf]

S1 Text

Mixtures. Crime-scene Y profiles can reflect a mixture of DNA from multiple male sources. It may be possible to identify the profiles underlying the mixture based on differing amounts of DNA or similarity to observed profiles, [1,2] but often they cannot be fully deconvolved with high confidence. We also performed simulations of a mixed Y profile with DNA from two male sources. For each of the three values of VRS and the Yfiler Plus profiling kit, we performed 10 population simulations, for each of these 10 mutation simulations were performed, and for each of these 1000 pairs of males were chosen at random to be the contributors to the mixture. For each contributor pair, the number of included live individuals was recorded, where "included" means has a Y profile consisting only of alleles observed in the mixture.

Among the included individuals will be those with profiles exactly matching one of the reference individuals. The sizes of these two sets of individuals will be approximately independent and have the same distribution as $|\Omega|$ for a single-source Y profile described above. S3 Fig. and S9 Table show that the number of included males who do not exactly match either reference individual is stochastically smaller than $|\Omega|$. Therefore the distribution of the total number of included males is conservatively estimated as the sum of three independent versions of $|\Omega|$. Further work is required to develop procedures for presenting evidence in court based on mixed Y profiles that takes available database information into account, but our simulations will provide helpful guidance.

References

1. Andersen MM, Eriksen PS, Morling N. The discrete Laplace exponential family and estimation of Y-STR haplotype frequencies. Journal of Theoretical Biology. 2013;329:39–51.

2. Andersen MM, Eriksen PS, Mogensen HS, Morling N. Identifying the most likely contributors to a Y-STR mixture using the discrete Laplace method. Forensic Science International: Genetics. 2015;15:76–83.
